# Demographics Associated With Stress, Severe Mental Distress, and Anxiety Symptoms During the COVID-19 Pandemic in Japan: Nationwide Cross-sectional Web-Based Survey

**DOI:** 10.2196/29970

**Published:** 2021-11-22

**Authors:** Haruhiko Midorikawa, Hirokazu Tachikawa, Takaya Taguchi, Yuki Shiratori, Asumi Takahashi, Sho Takahashi, Kiyotaka Nemoto, Tetsuaki Arai

**Affiliations:** 1 Graduate School of Comprehensive Human Sciences University of Tsukuba Tsukuba Japan; 2 Ibaraki Prefectural Medical Center of Psychiatry Kasama Japan; 3 Department of Disaster and Community Psychiatry University of Tsukuba Tsukuba Japan; 4 Tsukuba University Health Center University of Tsukuba Tsukuba Japan; 5 Department of Psychiatry University of Tsukuba Tsukuba Japan; 6 School of Humanities Hokusei Gakuen University Sapporo Japan

**Keywords:** COVID-19, mental health, stress, depression, anxiety, occupation, public health, demographic factors, epidemiology, occupational health

## Abstract

**Background:**

With the spread of COVID-19, the deterioration of public mental health has become a major global and social problem.

**Objective:**

The purpose of this study was to elucidate the relationship between the 3 mental health problems associated with COVID-19, that is, perceived stress, severe mental distress, and anxiety symptoms, and the various demographic factors, including occupation.

**Methods:**

A nationwide web-based questionnaire survey was conducted in Japan from August 4 to 31, 2020. In addition to sociodemographic data, the degrees of perceived stress, severe mental distress, and anxiety symptoms associated with COVID-19 were measured. After performing a descriptive statistical analysis, factors related to stress, severe mental distress, and anxiety symptoms were analyzed using logistic regression analysis.

**Results:**

A total of 8203 respondents submitted survey responses, among whom 34.9% (2861/8203) felt intense stress associated with COVID-19, 17.1% (1403/8203) were depressed, and 13.5% (1110/8203) had severe anxiety symptoms. The logistic regression analysis showed that each of the 3 mental health problems were prevalent in females, nonbinary gender, people in their 50s, 60s and older, respondents who visited psychiatrists, and those currently in psychiatric care. Severe mental distress and anxiety symptoms were associated with the number of effective lifestyle coping strategies during the lockdown period. Severe mental distress was only prevalent in teenagers and respondents in their 20s, as students tended to develop stress and severe mental distress. With regard to occupation, working in nursing care and welfare, education and research, and medical and health sectors was associated with stress; however, working in these occupations was not associated with severe mental distress and anxiety symptoms. Unemployment was associated with severe mental distress and anxiety symptoms. All 3 mental health problems were prevalent in part-time workers and those working in entertainment and arts sectors.

**Conclusions:**

Gender, age, occupation, history of psychiatric visits, and stress coping mechanisms were associated with mental health during the COVID-19 pandemic, but their associations with stress, severe mental distress, and anxiety symptoms differed. In addition, the actual state of mental health varied according to the respondents’ occupation. It is necessary to consider the impact of the COVID-19 pandemic on mental health not only at the individual level but also at the occupational level.

## Introduction

The global pandemic of the novel COVID-19 has continued since the unexplained advent of the pneumonia virus in Wuhan, China in December 2019 [1]. By the end of December 2020, 80 million cases were confirmed, with more than 1.7 million deaths globally [2]. Since then, various treatment methods, including vaccines, have been developed [3]; however, this unprecedented public health emergency of international concern has not yet been resolved. The effects of the COVID-19 pandemic are expected to continue in the future [4]. COVID-19 not only causes physical frailty but also gives rise to mental health problems [5]. Depression and anxiety have been reported to have increased among the public during the pandemic [6,7]. There are various causes of impaired mental health. Aside from anxiety regarding the disease, the policies employed by many countries to stop the spread of the infection (eg, quarantine, social distancing, isolation of infected people) have also affected people’s mental health [8,9]. One of the main causes is the economic deterioration due to the stagnation of socioeconomic activities [10].

Studies related to mental health during the COVID-19 pandemic have gradually increased and continue to do so. Although there is a high degree of heterogeneity in studies of mental health during the COVID-19 pandemic due to differences in the situation of the infection in different countries, on average, approximately 30% of the general population have reported depressive and anxiety symptoms [11,12]. In a survey conducted in Japan in May 2020, 11.5% of the respondents reported serious psychological distress [13]. This may be because the number of infected people in Japan at that time was lower than that in the Western countries [2]. At the end of August 2020, when this survey was conducted, the number of people infected with COVID-19 in Japan was approximately 70,000, which was still lower than that in the Western countries [2]. However, the number of suicides in Japan started increasing from that period [14], and mental health issues related to COVID-19 have now become a major issue in Japan.

In previous studies, various factors such as gender, age, marital status, education, occupation and income, place of residence, contact history with patients with COVID-19, and comorbidities were associated with mental health problems such as stress, depression, and anxiety [15-18]. However, in many studies, stress has been measured with a general psychological scale, which differs from the perceived stress scale associated with COVID-19 [6,11]. Therefore, the relationship between various factors and each mental health problem related to COVID-19 such as perceived stress, depression, and anxiety has not been sufficiently investigated. Moreover, although the COVID-19 pandemic has had a great impact on society and people's behavior, there is a lack of occupational research on the same. Previous studies have often focused on specific occupations such as health care professionals and employment status [17]; however, a cross-sectional evaluation of mental health with respect to various occupations and considering it in combination with other attributes has not been conducted. Therefore, we conducted a nationwide web-based questionnaire survey in Japan to determine the relationship between various factors, including occupation, and perceived stress, severe mental distress, and anxiety symptoms, which are the 3 main mental health problems related to the spread of the COVID-19 pandemic.

## Methods

### Recruitment

From August 4 to August 31, 2020, we conducted a nationwide web-based questionnaire survey in Japan by using a web-based survey platform called SurveyMonkey [19]. Respondents were recruited through various news and social media sites such as Facebook and Twitter. Specifically, in addition to the snowball sampling method utilized by the researchers, announcements were made on the official websites of the laboratory and the University of Tsukuba. Additionally, major news media reported that this research was being conducted. The survey questionnaire was distributed among respondents who provided their informed consent after reading about the purpose of the survey on the first page and clicking on the check boxes. The survey was conducted anonymously, and no personally identifiable information was included in the data used for analysis. Furthermore, no exclusion criteria were established. Missing values for the social parameters are shown for each item. This study was approved by the Medical Ethics Committee of the University of Tsukuba (Registration 1546-1). Appropriate ethical considerations were applied during all the stages of this study.

### Measures

We asked the respondents about their gender, age, occupation, and psychiatric outpatient history. We also enquired whether they experienced stress related to COVID-19 in the previous month. The response options included “strongly disagree,” “disagree,” “neither agree nor disagree,” “agree,” “strongly agree,” and “other.” We categorized those who chose the option “strongly agree” as those under severe stress related to COVID-19. Another question inquired whether the respondents felt that they or their families were at risk of infection, whether they were bullied or discriminated against, and whether self-confinement interfered with work or school. We also investigated the number of effective lifestyle coping strategies employed during the self-confinement period, such as maintaining a healthy rhythm of life, exercise, good eating habits, getting enough sleep, enjoying indoor activities, talking to friends, encouraging each other, reducing the amount of time spent watching television or surfing the internet, and getting the right information on COVID-19. The Japanese version of the 6-item Kessler Psychological Distress Scale (K6) and Generalized Anxiety Disorder-7 item (GAD-7) were used to measure severe mental distress and anxiety symptoms.

The K6 is a short, 6-item questionnaire developed for screening mood and anxiety disorders [20]. It evaluates mood and anxiety experienced in the last 30 days on a 5-point scale (ranging from 0 to 4). The total score for K6 ranged from 0 to 24. We used the Japanese version of K6, which has demonstrated confirmed reliability and validity [21]. Based on Kessler et al’s recommendation [20], we adopted K6≥13 as the cut-off value, indicating a state of severe mental distress.

The GAD-7 is a self-administered questionnaire developed by Spitzer et al to assess the severity of generalized anxiety disorder by extracting a response set from the Public Health Questionnaire [22]. It consists of 7 items and evaluates the intensity of symptoms experienced in the past 2 weeks on a 4-point scale (0 to 3). The total score for GAD-7 ranged from 0 to 21. We used the Japanese version of GAD-7, which has exhibited robust reliability and validity [23]. The severity of the anxiety symptoms based on the GAD-7 score was assessed as follows: a score of 5-9 points indicated mild anxiety, 10-14 points indicated moderate anxiety, and ≥15 points indicated severe anxiety. We adopted GAD-7≥10 as the cut-off value indicating a state of anxiety symptoms.

### Statistical Analysis

We first confirmed the response status of all respondents. Following this, we verified those who experienced severe stress related to COVID-19, those who were depressed (K6 score≥13), and those who had severe anxiety symptoms (GAD-7 score≥10). For these 3 indicators, we then excluded respondents with missing values and performed a chi-square test with Bonferroni correction (using the Bonferroni correction, .05 is divided by the number of statistical tests being performed, .05/15=0.00333; therefore, *P*<.003). Additionally, a residual analysis was carried out to see how the various factors, including occupation type, are interrelated. An adjusted standardized residual >3.29 or <–3.29 was considered significant (*P*<.001). Then, a logistic regression analysis was performed with each psychological indicator as a dependent variable and demographic factors, including occupation type, as independent variables (statistical significance was defined as *P*<.05). Variables that were reported to be associated with mental health during the COVID-19 pandemic, in previous studies, were included in the model. Nagelkerke’s R^2^ was calculated to check the goodness of fit of the logistic regression model. All statistical analyses were performed using the statistical package SPSS Statistics for Windows, version 22.0 (IBM Corp).

## Results

### Characteristics of the Respondents

The total number of consenting respondents was 8203. Table 1 lists the attributes of all respondents. Of the 8203 total responses, 4692 responses were from females. The age group was widely distributed from teenagers to those older than 60 years, but only 3.9% (324/8203) of the total respondents were aged 60 years and older. Unemployed people comprised 11.8% (971/8203) of the total respondents, and medical and health professionals comprised 11.7% (960/8203) of the employed people. Approximately 34.9% (2861/8203) of the respondents reported that they had been very stressed about COVID-19 over the previous month. During the spread of COVID-19, 52.8% (4333/8203) felt that they were at risk of infection, 46.4% (3808/8203) felt hindered by the lockdown, and 2.1% (175/8203) were bullied or discriminated against because they worked at a hospital that treats patients with COVID-19, which may have exposed them to the risk of infection.

**Table 1:** Characteristics of the respondents of a nationwide web-based survey that was conducted to examine the relationship between the COVID-19 pandemic and mental health in Japan in 2020 (N=8203).

Characteristic		Values, n (%)
**Gender**		
	Female	4692 (57.2)
	Male	2700 (32.9)
	Nonbinary	128 (1.6)
	Unknown	683 (8.3)
**Age group (years)**		
	Teenage	178 (2.2)
	20-29	1543 (18.8)
	30-39	2106 (25.7)
	40-49	2073 (25.3)
	50-59	1296 (15.8)
	≥60	324 (3.9)
	Unknown	683 (8.3)
**Occupation**		
	Unemployed	971 (11.8)
	Medical and health	960 (11.7)
	Education and research	754 (9.2)
	Student	681 (8.3)
	Information and communication systems	639 (7.8)
	Other	531 (6.5)
	Part-time job	494 (6)
	Manufacturer	444 (5.4)
	Entertainment and arts	415 (5.1)
	Nursing care and welfare	392 (4.8)
	Government job	343 (4.2)
	Sales and wholesale	343 (4.2)
	Infrastructure and construction	165 (2)
	Finance and insurance	142 (1.7)
	Food, beverage, and accommodation	96 (1.2)
	Transportation	79 (1)
	Agriculture, forestry, and fisheries	32 (0.4)
	Unknown	722 (8.8)
**History of psychiatric visits**		
	Currently going to the hospital	1148 (14)
	History of hospital visit(s)	1823 (22.2)
	None	4420 (53.9)
	Other/unknown	812 (9.9)
**Degree of stress associated with COVID-19**		
	Strongly agree	2861 (34.9)
	Agree	3143 (38.3)
	Neither agree nor disagree	286 (3.5)
	Disagree	963 (11.7)
	Strongly disagree	194 (2.4)
	Other	756 (9.2)
**Severe mental distress**		
	K6^a^≥13	1403 (17.1)
	K6<13	5877 (71.6)
	Unknown	923 (11.3)
**Anxiety symptoms**		
	GAD-7^b^≥10	1127 (13.7)
	GAD-7<10	5966 (72.7)
	Unknown	1110 (13.5)
**Experience during the spread of the COVID-19 infection**		
	Felt at risk of infection: yes	4333 (52.8)
	Felt at risk of infection: no	3187 (38.9)
	Felt at risk of infection: unknown	683 (8.3)
	Hindered by the lockdown: yes	3808 (46.4)
	Hindered by the lockdown: no	3712 (45.3)
	Hindered by the lockdown: unknown	683 (8.3)
	Being bullied or discriminated against: yes	175 (2.1)
	Being bullied or discriminated against: no	7345 (89.5)
	Being bullied or discriminated against: unknown	683 (8.3)
**Number of effective lifestyle coping mechanisms employed during lockdown**		
	0	274 (3.3)
	1-3	1934 (23.6)
	4-6	2872 (35)
	7-10	1467 (17.9)
	Unknown	1656 (20.2)

^a^K6: 6-item Kessler Psychological Distress Scale.

^b^GAD-7: Generalized Anxiety Disorder-7 item.

### Perceived Stress, Anxiety Symptoms, and Severe Mental Distress

Respondents without missing responses were divided into groups according to whether they experienced severe stress associated with the COVID-19 pandemic (excluding those who answered “other” on this question, 6363/8203, 77.6% of the respondents), whether they experienced severe mental distress (6424/8203, 78.3% of the respondents), and whether they experienced anxiety symptoms (6424/8203, 78.3% of the respondents). Tables 2, 3, and 4 present the characteristics of each group. In terms of gender, female respondents experienced the most COVID-19–related stress, whereas severe mental distress and anxiety symptoms were the highest in nonbinary respondents. According to age group, COVID-19–related stress was the highest among respondents in their 50s, whereas severe mental distress was the highest among teenagers, and anxiety symptoms were the highest among people in their 20s. Regarding occupation, stress was particularly high for those working in the medical and health fields. Furthermore, severe mental distress was the most common among the unemployed and students. Severe anxiety symptoms were also most commonly reported by the unemployed. Regarding the history of psychiatric visits, those who were currently seeing a psychiatrist comprised the highest percentages of those who reported severe stress, severe mental distress, and anxiety symptoms.

**Table 2:** Characteristics of those who reported experiencing COVID-19–related severe stress in the nationwide web-based survey that was conducted to examine the relationship between the COVID-19 pandemic and mental health in Japan in 2020.

Variables			Severe stress related to COVID-19					
			Yes (n=2443), n (%)		No (n=3920), n (%)		*P* value^a^	
**Gender**								<.001
	Male	683 (30.1)^b^		1589 (69.9)^b^				
	Female	1717 (43.1)^b^		2265 (56.9)^b^				
	Nonbinary	43 (39.4)		66 (60.6)				
**Age (years)**								<.001
	Teenage	36 (27.9)		93 (72.1)				
	20-29	452 (35.5)		823 (64.5)				
	30-39	655 (36.7)		1129 (63.3)				
	40-49	689 (38.4)		1103 (61.6)				
	50-59	490 (44.1)^b^		622 (55.9)^b^				
	≥60	121 (44.6)		150 (55.4)				
**Occupation**								<.001
	Information and communication systems	150 (27.5)^b^		396 (72.5)^b^				
	Agriculture, forestry, and fisheries	9 (29)		22 (71)				
	Transportation	20 (29.4)		48 (70.6)				
	Student	182 (32)^b^		387 (68)^b^				
	Manufacturer	122 (32.3)		256 (67.7)				
	Government	100 (33.4)		199 (66.6)				
	Unemployed	291 (37)		495 (63)				
	Part-time job	160 (39.1)		249 (60.9)				
	Finance and insurance	45 (37.2)		76 (62.8)				
	Sales and wholesale	108 (37.5)		180 (62.5)				
	Infrastructure and construction	59 (39.6)		90 (60.4)				
	Food, beverage, and accommodation	28 (36.4)		49 (63.6)				
	Other	179 (39.3)		277 (60.7)				
	Entertainment and arts	149 (42.1)		205 (57.9)				
	Nursing care and welfare	149 (44.2)		188 (55.8)				
	Education and research	289 (44.2)		365 (55.8)				
	Medical and health	403 (47.9)^b^		438 (52.1)^b^				
**History of psychiatric visits**								<.001
	None	1392 (36.4)^b^		2429 (63.6)^b^				
	History of hospital visit(s)	620 (39.7)		943 (60.3)				
	Currently going to the hospital	431 (44)^b^		548 (56)^b^				
**Number of coping mechanisms employed during self-confinement**								.56
	0-3	827 (38.4)		1326 (61.6)				
	4-6	1054 (37.8)		1734 (62.2)				
	7-10	562 (39.5)		860 (60.5)				

^a^With Bonferroni correction for the 15 tests, the threshold *P* value for significance was <.003.

^b^Significant at *P*<.001.

**Table 3:** Characteristics of those who reported experiencing severe mental distress in the nationwide web-based survey that was conducted to examine the relationship between the COVID-19 pandemic and mental health in Japan in 2020.

Variables		Severe mental distress (6-item Kessler Psychological Distress Scale≥13)			
		Yes (n=1215), n (%)	No (n=5209), n (%)	*P* value^a^	
**Gender**					<.001
	Male	336 (14.6)^b^	1958 (85.4)^b^		
	Female	824 (20.5)^b^	3196 (79.5)^b^		
	Nonbinary	55 (50)^b^	55 (50)^b^		
**Age (years)**					<.001
	Teenage	42 (32.3)^b^	88 (67.7)^b^		
	20-29	356 (27.7)^b^	927 (72.3)^b^		
	30-39	378 (21)	1421 (79)		
	40-49	285 (15.7)^b^	1527 (84.3)^b^		
	50-59	137 (12.2)^b^	986 (87.8)^b^		
	≥60	17 (6.1)^b^	260 (93.9)^b^		
**Occupation**					<.001
	Information and communication systems	85 (15.4)	466 (84.6)		
	Agriculture, forestry, and fisheries	2 (6.5)	29 (93.5)		
	Transportation	14 (20.6)	54 (79.4)		
	Student	164 (28.7)^b^	407 (71.3)^b^		
	Manufacturer	65 (17.1)	315 (82.9)		
	Government	53 (17.7)	246 (82.3)		
	Unemployed	231 (28.9)^b^	568 (71.1)^b^		
	Part-time job	93 (22.6)	319 (77.4)		
	Finance and insurance	17 (14)	104 (86)		
	Sales and wholesale	56 (19.4)	233 (80.6)		
	Infrastructure and construction	28 (18.8)	121 (81.2)		
	Food, beverage, and accommodation	9 (11.5)	69 (88.5)		
	Other	79 (16.8)	391 (83.2)		
	Entertainment and arts	88 (24.7)	268 (75.3)		
	Nursing care and welfare	58 (17)	284 (83)		
	Education and research	93 (14.1)^b^	566 (85.9)^b^		
	Medical and health	80 (9.4)^b^	769 (90.6)^b^		
**History of psychiatric visits**					<.001
	None	469 (12.2)^b^	3387 (87.8)^b^		
	History of hospital visit(s)	321 (20.3)	1259 (79.7)		
	Currently going to the hospital	425 (43)^b^	563 (57)^b^		
**Number of coping mechanisms employed during self-confinement**					<.001
	0-3	544 (25.1)^b^	1620 (74.9)^b^		
	4-6	491 (17.4)	2329 (82.6)		
	7-10	180 (12.5)^b^	1260 (87.5)^b^		

^a^With Bonferroni correction for the 15 tests, the threshold *P* value for significance was <.003.

^b^Significant at *P*<.001.

**Table 4:** Characteristics of those who reported experiencing anxiety symptoms in the nationwide web-based survey conducted that was to examine the relationship between the COVID-19 pandemic and mental health in Japan in 2020.

Variables		Anxiety symptoms (Generalized Anxiety Disorder-7 item≥10)			
		Yes (n=996), n (%)	No (n=5428), n (%)	*P* value^a^	
**Gender**					<.001
	Male	308 (13.4)^b^	1986 (86.6)^b^		
	Female	646 (16.1)	3374 (83.9)		
	Nonbinary	42 (38.2)^b^	68 (61.8)^b^		
**Age (years)**					<.001
	Teenage	22 (16.9)	108 (83.1)		
	20-29	241 (18.8)^b^	1042 (81.2)^b^		
	30-39	317 (17.6)	1482 (82.4)		
	40-49	242 (13.4)	1570 (86.6)		
	50-59	153 (13.6)	970 (86.4)		
	≥60	21 (7.6)^b^	256 (92.4)^b^		
**Occupation**					<.001
	Information and communication systems	64 (11.6)	487 (88.4)		
	Agriculture, forestry, and fisheries	1 (3.2)	30 (96.8)		
	Transportation	13 (19.1)	55 (80.9)		
	Student	100 (17.5)	471 (82.5)		
	Manufacturer	51 (13.4)	329 (86.6)		
	Government	41 (13.7)	258 (86.3)		
	Unemployed	198 (24.8)^b^	601 (75.2)^b^		
	Part-time job	86 (20.9)	326 (79.1)		
	Finance and insurance	13 (10.7)	108 (89.3)		
	Sales and wholesale	45 (15.6)	244 (84.4)		
	Infrastructure and construction	27 (18.1)	122 (81.9)		
	Food, beverage, and accommodation	6 (7.7)	72 (92.3)		
	Other	76 (16.2)	394 (83.8)		
	Entertainment and arts	69 (19.4)	287 (80.6)		
	Nursing care and welfare	49 (14.3)	293 (85.7)		
	Education and research	85 (12.9)	574 (87.1)		
	Medical and health	72 (8.5)^b^	777 (91.5)^b^		
**History of psychiatric visits**					<.001
	None	351 (9.1)^b^	3505 (90.9)^b^		
	History of hospital visit(s)	278 (17.6)	1302 (82.4)		
	Currently going to the hospital	367 (37.1)^b^	621 (62.9)^b^		
**Number of coping mechanisms employed during self-confinement**					<.001
	0-3	437 (20.2)^b^	1727 (79.8)^b^		
	4-6	396 (14)	2424 (86)		
	7-10	163 (11.3)^b^	1277 (88.7)^b^		

^a^With Bonferroni correction for the 15 tests, the threshold *P* value for significance was <.003.

^b^Significant at *P*<.001.

### Logistic Regression Analysis of Perceived Stress, Severe Mental Distress, and Anxiety Symptoms

Tables 5, 6, and 7 show the results of the logistic regression analysis for each mental health problem. Women, nonbinary people, part-time workers, those working in entertainment and arts, people who had visited a psychiatrist in the past, and those who were currently under psychiatric care were more likely to have these mental health problems. Teenagers (*P*=.01) and people in their 20s (*P*<.001) exhibited a significantly higher probability of experiencing severe mental distress, and people in their 50s (*P*<.001) and 60s and older (*P*<.003) exhibited a significantly higher probability of experiencing severe stress related to COVID-19. In contrast, people in their 40s, 50s, and 60s and older exhibited a significantly lower probability of experiencing severe mental distress (*P*<.001) or feeling anxious (40s: *P*=.004; 50s: *P*=.04; 60s and older: *P*=.002).

In terms of occupation, respondents who felt highly stressed were more likely to be students (*P*=.03) and those working in sales and wholesale (*P*=.01), infrastructure and construction (*P*=.01), nursing and welfare (*P*<.001), education and research (*P*<.001), and medical and health sectors (*P*<.001). Students also exhibited a significantly higher probability of experiencing severe mental distress (*P*=.01), and people in sales and wholesale (*P*=.04) and infrastructure and construction (*P*=.02) also exhibited a significantly higher probability of feeling anxious. However, although unemployed persons did not exhibit a significantly higher probability of experiencing severe stress, they were highly likely to experience severe mental distress and anxiety symptoms (*P*<.001). Moreover, those who had employed effective lifestyle coping mechanisms during the lockdown period exhibited a significantly lower probability of experiencing severe mental distress or feeling anxious (*P*<.001).

**Table 5:** Logistic regression analysis of the COVID-19–related severe stress that was reported in a nationwide web-based survey that was conducted to examine the relationship between the COVID-19 pandemic and mental health in Japan in 2020.^a^

Variable			Severe stress related to COVID-19			
			Odds ratio (95% CI)		*P* value	
**Gender**						<.001
	Male	1.000		N/A^b^		
	Female	1.693 (1.512-1.895)		<.001		
	Other	1.496 (1.002-2.236)		.049		
**Age (years)**						<.001
	Teenage	0.768 (0.492-1.198)		.24		
	20-29	1.034 (0.875-1.222)		.69		
	30-39	1.000		N/A		
	40-49	1.075 (0.936-1.234)		.31		
	50-59	1.335 (1.142-1.561)		<.001		
	≥60	1.505 (1.153-1.966)		.003		
**Occupation**						<.001
	Information and communication systems	1.000		N/A		
	Agriculture, forestry, and fisheries	1.190 (0.530-2.674)		.67		
	Transportation	1.171 (0.669-2.052)		.58		
	Student	1.368 (1.025-1.824)		.03		
	Manufacturer	1.307 (0.978-1.746)		.07		
	Government	1.278 (0.938-1.740)		.12		
	Unemployed	1.221 (0.956-1.560)		.11		
	Part-time job	1.399 (1.060-1.848)		.02		
	Finance and insurance	1.361 (0.895-2.069)		.02		
	Sales and wholesale	1.475 (1.084-2.007)		.01		
	Infrastructure and construction	1.660 (1.132-2.435)		.01		
	Food, beverage, and accommodation	1.301 (0.784-2.158)		.31		
	Other	1.486 (1.134-1.946)		.004		
	Entertainment and art	1.768 (1.327-2.354)		<.001		
	Nursing care and welfare	1.828 (1.368-2.443)		<.001		
	Education and research	1.946 (1.519-2.494)		<.001		
	Medical and health	2.313 (1.826-2.929)		<.001		
**History of psychiatric visits**						<.001
	None	1.000		N/A		
	History of hospital visit	1.186 (1.047-1.343)		.007		
	Currently going to the hospital	1.507 (1.296-1.751)		<.001		
Number of effective coping mechanisms employed during self-confinement			0.986 (0.965-1.009)		.24	

^a^Nagelkerke’s *R^2^*=0.050.

^b^N/A: not applicable.

**Table 6:** Logistic regression analysis of the severe mental distress reported in a nationwide web-based survey that was conducted to examine the relationship between the COVID-19 pandemic and mental health in Japan in 2020.^a^

Variable		Severe mental distress (6-item Kessler Psychological Distress Scale≥13), n (%)		
		Odds ratio (95% CI)	*P* value	
**Gender**				<.001
	Male	1.000	N/A^b^	
	Female	1.652 (1.421-1.922)	<.001	
	Other	4.135 (2.700-6.333)	<.001	
**Age (years)**				<.001
	Teenage	1.780 (1.124-2.821)	.01	
	20-29	1.452 (1.194-1.767)	<.001	
	30-39	1.000	N/A	
	40-49	0.724 (0.604-0.868)	<.001	
	50-59	0.529 (0.423-0.663)	<.001	
	≥60	0.291 (0.173-0.491)	<.001	
**Occupation**				<.001
	Information and communication systems	1.000	N/A	
	Agriculture, forestry, and fisheries	0.467 (0.106-2.049)	.31	
	Transportation	1.604 (0.823-3.123)	.17	
	Student	1.550 (1.104-2.174)	.01	
	Manufacturer	1.359 (0.936-1.972)	.11	
	Government	1.167 (0.785-1.735)	.45	
	Unemployed	1.731 (1.282-2.337)	<.001	
	Part-time job	1.428 (1.007-2.026)	.046	
	Finance and insurance	0.818 (0.452-1.481)	.51	
	Sales and wholesale	1.472 (0.994-2.181)	.054	
	Infrastructure and construction	1.369 (0.833-2.250)	.22	
	Food, beverage, and accommodation	0.769 (0.362-1.633)	.49	
	Other	1.184 (0.830-1.689)	.35	
	Entertainment and art	1.856 (1.299-2.651)	.001	
	Nursing care and welfare	1.124 (0.764-1.655)	.55	
	Education and research	1.159 (0.827-1.624)	.39	
	Medical and health	0.717 (0.508-1.010)	.06	
**History of psychiatric visits**				<.001
	None	1.000	N/A	
	History of hospital visit	1.794 (1.524-2.111)	<.001	
	Currently going to the hospital	5.006 (4.215-5.945)	<.001	
Number of effective coping mechanisms employed during self-confinement		0.869 (0.843-0.895)	<.001	

^a^Nagelkerke’s *R^2^*=0.192.

^b^N/A: not applicable.

**Table 7:** Logistic regression analysis of the anxiety symptoms reported in a nationwide web-based survey that was conducted to examine the relationship between the COVID-19 pandemic and mental health in Japan in 2020.^a^

Variable			Anxiety symptoms (Generalized Anxiety Disorder-7 item≥10)		
			Odds ratio (95% CI)		*P* value
**Gender**					<.001
	Male	1.000		N/A^b^	
	Female	1.233 (1.054-1.442)		.009	
	Other	2.694 (1.746-4.157)		<.001	
**Age (years)**					<.001
	Teenage	1.083 (0.624-1.880)		.78	
	20-29	1.127 (0.910-1.396)		.27	
	30-39	1.000		N/A	
	40-49	0.753 (0.622-0.912)		.004	
	50-59	0.789 (0.632-0.984)		.04	
	≥60	0.471 (0.292-0.759)		.002	
**Occupation**					<.001
	Information and communication systems	1.000		N/A	
	Agriculture, forestry, and fisheries	0.310 (0.041-2.344)		.26	
	Transportation	1.933 (0.975-3.829)		.06	
	Student	1.439 (0.982-2.109)		.06	
	Manufacturer	1.360 (0.905-2.044)		.14	
	Government	1.212 (0.784-1.874)		.39	
	Unemployed	1.866 (1.350-2.581)		<.001	
	Part-time job	1.809 (1.249-2.619)		.002	
	Finance and insurance	0.871 (0.453-1.675)		.68	
	Sales and wholesale	1.576 (1.028-2.417)		.04	
	Infrastructure and construction	1.803 (1.082-3.033)		.02	
	Food, beverage, and accommodation	0.698 (0.287-1.693)		.43	
	Other	1.524 (1.049-2.214)		.03	
	Entertainment and art	1.896 (1.287-2.794)		.001	
	Nursing care and welfare	1.253 (0.827-1.899)		.29	
	Education and research	1.381 (0.963-1.981)		.08	
	Medical and health	0.902 (0.624-1.303)		.58	
**History of psychiatric visits**					<.001
	None	1.000		N/A	
	History of hospital visit	2.038 (1.713-2.425)		<.001	
	Currently going to the hospital	5.133 (4.293-6.136)		<.001	
Number of effective coping mechanisms employed during self-confinement			0.898 (0.871-0.927)		<.001

^a^Nagelkerke’s R^2^=0.147.

^b^N/A: not applicable.

## Discussion

In this study, we conducted a nationwide web-based questionnaire survey in Japan to shed light on the association between 3 mental health problems related to the COVID-19 pandemic (perceived stress, severe mental distress, and anxiety symptoms) and various factors. The strength of this study is that perceived stress was evaluated subjectively in relation to the COVID-19 pandemic and that various occupations were taken into consideration in evaluating the same. A large portion of the sample experienced stress, severe mental distress, and anxiety symptoms. However, their characteristics and experiences were associated with these problems differently; occupation, in particular, seemed to have a significant impact on these differences. Approximately 73.2% (6004/8203) of the respondents experienced perceived stress related to the COVID-19 pandemic, and 34.9% (2861/8203) of the respondents experienced severe stress. In addition, 17.1% (1403/8203) of the respondents had a score of 13 or higher on the K6 depression scale, and 13.7% (1127/8203) had a score of 10 or higher on the GAD-7 anxiety scale. In a similar study conducted in Japan, 11.5% of the respondents had a K6 score of 13 or higher [13]; prior to this survey being conducted in April 2020, the Government of Japan declared a state of emergency in response to the first wave of the pandemic, and some prefectures were put on a mild lockdown. Given that this study was conducted during the second wave, wherein a subsequent state of emergency was not declared, it is not possible to make a simple comparison due to the difference in conditions. However, due to the prolongation of the COVID-19 pandemic, it has been suggested that the accompanying lifestyle changes may have an adverse effect on mental health. Nevertheless, compared to other countries, the proportion of people in Japan with severe mental distress was not high [24,25]. A similar trend was observed with regard to anxiety [26-28]. It is believed that these tendencies were because the spread of the infection was relatively controlled in Japan compared to other countries at that time.

The results of logistic regression analyses revealed that perceived stress, severe mental distress, and anxiety symptoms related to the COVID-19 pandemic are associated with factors such as gender, age, occupation, history of psychiatric visits, and stress-related coping mechanisms. Factors that have a common negative association with these 3 mental health problems include being a woman and having a history of psychiatric visits. Regarding age, the higher the age, the higher the COVID-19–related stress, whereas severe mental distress and anxiety symptoms were more severe in younger people. Furthermore, the existence of coping mechanisms contributed to the reduction in severe mental distress and anxiety symptoms. These associations have also been reported in previous studies [13,26,29-31].

In terms of occupation, different associations with perceived stress, severe mental distress, and anxiety symptoms that are associated with the COVID-19 pandemic were identified. Since people from the information and communication systems industry experienced relatively good mental health, they were used as a standard for making comparisons. With the spread of COVID-19, the information and communication systems sector has become even more important in the society [32,33]. It has also been reported that in Japan, the rate of people working from home in the telecommunications industry is higher than that for other occupations [34]. These factors may have positively influenced their mental health.

Meanwhile, students had high levels of stress and severe mental distress. Previous studies have also revealed that COVID-19 has adversely affected students’ mental health [35]. In Japan, most schools are forced to close during emergencies. Subsequently, during the lockdown due to the COVID-19 pandemic, classes were conducted online [36]. Student suicide is known to be common at the beginning of the semester [37,38]. These results, coupled with the increase in suicide rate among students since the spread of the second wave of the COVID-19 infection in Japan [39], may have influenced the new web-based school setup during the pandemic. They comprised the category most hindered by the lockdown (see Multimedia Appendix 1).

After students, those from the sectors of the food, beverage, and accommodation; entertainment and arts; education and research; and part-time work comprised a large proportion of people who experienced stress due to the lockdown, though to varied degrees (see Multimedia Appendix 1). The food, beverage, and accommodation industry was one of the sectors that was significantly affected by the COVID-19 pandemic [40], and many felt that they were at risk of infection; however, there were no significant differences in stress (*P*=.31), severe mental distress (*P*=.49), or anxiety symptoms (*P*=.43). This may have been because the Japanese government initiated focused financial support for the food, beverage, and accommodation industry during the survey period [41], though it cannot be denied that this may also be because a relatively small number of people were affected. There were no significant differences in severe mental distress and anxiety symptoms among people working in the field of education and research. This may have been influenced by the fact that relatively few of them felt that they were at risk of infection during the period when this survey was conducted. Meanwhile, the entertainment and arts sectors reported high levels of perceived stress, severe mental distress, and anxiety symptoms. The COVID-19 pandemic was particularly devastating to these sectors due to the rules on social distancing in many countries, the ban on large-scale gatherings, and the need to refrain from going out for nonessential reasons [42,43]. In addition, the news in Japan about a celebrity’s suicide that took place a month before the survey may have had an impact [44]. Furthermore, high levels of perceived stress, severe mental distress, and anxiety symptoms among people working in part-time jobs may be due to their unstable employment and vulnerable positions [45-47].

With regard to occupations such as sales and wholesale as well as infrastructure and construction, the percentage of those who responded that there was a problem due to the lockdown was lower than that in the industries listed above, but perceived stress and anxiety symptoms were higher. Although there was no significant difference, depressive tendencies were also observed. COVID-19 caused a chain of economic impacts in each sector due to supply chain turmoil; this result is believed to be a reflection of those impacts [48]. In the unemployed group, no increase in perceived stress associated with COVID-19 was observed, but severe mental distress and anxiety symptoms were high. We cannot rule out the possibility that the unemployed individuals in our study were unemployed before the spread of COVID-19, and it is possible that they had mental health problems regardless of COVID-19. A previous study reported that unemployment is associated with depression and reduced self-esteem [49]. However, due attention should be paid to the results of this study, as previous studies have also identified unemployment as a risk factor of depression during the COVID-19 pandemic [50].

Nursing care, welfare, medical care, and health care sectors were associated with increased stress due to COVID-19. Since these professions tend to confront COVID-19 directly, it is natural for people working in these sectors to feel the risk of infection and feel highly stressed. In addition, since prejudice and discrimination against health care workers have become a problem [51], the proportion of health care professionals who complained of discrimination was also large in this study. Interestingly, this was not associated with high levels of severe mental distress or anxiety symptoms in this study. There have been numerous reports of deterioration in the mental health of health care professionals during the COVID-19 pandemic [52]. The inconsistency between the results of this study and those of previous studies may be because this study included a small percentage of respondents who were actually engaged in the treatment of patients with COVID-19 [53]. This study did not collect information on whether respondents were infected with COVID-19 or had contact with patients with COVID-19. Our results may also have been affected by the fact that infection was relatively controlled in Japan compared to other countries at the time of the survey. Additionally, having adequate knowledge of COVID-19 [54,55] may also prevent the exacerbation of depression and anxiety.

The limitations of our survey are as follows. First, since it was a web-based questionnaire survey that allowed free participation, sampling bias and the effect of duplicate responses and missing values must be considered. We confirmed that responses were obtained from a wide range of people based on demographic factors such as occupation; however, the groups do not represent the general Japanese population. Second, although the spread of infection changes daily and varies across regions, we did not account for the impact of this in the study. Third, the details regarding the respondents’ occupation, such as whether or not they worked from home, are not clear; therefore, its impact cannot be evaluated. Past studies have reported that worrying about not being able to work from home is associated with poor mental health [56]. Fourth, the actual economic status of each respondent was unknown. Economic status also affects mental health [57]. Fifth, since we did not inquire whether the respondents had ever been infected with COVID-19, we do not know the effect of the respondents’ personal experiences with past infection on their mental health. Sixth, this study focused on perceived stress; however, it did not examine posttraumatic stress. Finally, it is difficult to evaluate the causal relationships since this study adopted a cross-sectional design.

Despite these limitations, the study found that COVID-19–related perceived stress, severe mental distress, and anxiety symptoms—the 3 mental health-related issues—tended to differ by occupation. These gaps indicate the complexity of changes in people’s mental health during the COVID-19 pandemic. These results suggest that there may be 2 main mechanisms underlying mental health problems. One is the direct deterioration of mental health due to the accumulation of stress associated with COVID-19, which is represented by the remarkably high stress in the medical and health professions. This may include fear of infection and overwork in industries where the workload increased with the spread of infection. Burnout among health care workers has become a problem in Western countries, where the number of people infected with COVID-19 is high [58], and priority measures are needed to reduce COVID-19–related stress. The other is the indirect deterioration of mental health as a result of changes in the socioeconomic conditions due to COVID-19. As COVID-19 has become a more prolonged problem than natural disasters, changes in social structure and economic conditions have become more serious and uncertainty about the future is continually increasing. In our study, occupations with high COVID-19–related stress did not necessarily coincide with those experiencing severe mental distress. Although some degree of stress is assumed, it is suggested that those who are socially unstable or vulnerable to change may develop severe mental distress due to the indirect effects of the COVID-19 pandemic. As the second wave of the COVID-19 infection gradually subsides, these people may be excluded from the process of resuming economic and social activities, face severe realities, and may be exposed to a high risk of suicide. To prevent the increase in suicide cases caused by the COVID-19 pandemic, it is ideal to implement socioeconomic and mental health measures focusing on groups that experience high levels of severe mental distress and anxiety symptoms, as pointed out in this study.

In conclusion, gender, age, occupation, history of psychiatric visits, and stress-coping mechanisms were associated with mental health during the COVID-19 pandemic. In particular, in terms of occupations, a strong association with severe mental distress was noted in students, unemployed individuals, part-time workers, and people working in the entertainment and arts industry. Since mental health problems differ depending on the type of occupation, combatting the adverse effects of the COVID-19 pandemic requires more active socioeconomic and preventive mental health measures for those in fields/occupations that have been affected the most by the pandemic. In addition, it will be necessary to conduct further detailed research to clarify how the COVID-19 pandemic causes mental health problems not only at the individual level but also at the occupational level.

## Appendix

Multimedia Appendix 1 Differences in the experiences during the spread of the COVID-19 infection by occupation in a nationwide web-based survey that was conducted to examine the relationship between the COVID-19 pandemic and mental health in Japan in 2020.

## Abbreviations

GAD-7: Generalized Anxiety Disorder-7 item
K6: 6-item Kessler Psychological Distress Scale
