# Supplementary material for: Demographics Associated With Stress, Severe Mental Distress, and Anxiety Symptoms During the COVID-19 Pandemic in Japan: Nationwide Cross-sectional Web-Based Survey
Source: JMIR Public Health Surveill. 2021 Nov 22;7(11):e29970. doi: 10.2196/29970 (PMC8610490; doi:10.2196/29970)
Supplement: Multimedia Appendix 1 [file publichealth_v7i11e29970_app1.docx]

Multimedia Appendix 1. Differences in the experiences during the spread of the COVID-19 infection, by occupation in a nationwide online survey that was conducted to examine the relationship between the COVID-19 pandemic and mental health in Japan in 2020.

| Occupation | Felt at risk of infection,  n (%) | Hindered due to lockdown,  n (%) | Experienced bullying or discrimination,  n (%) |
| --- | --- | --- | --- |
| Total | 3730 (58.1) | 3280 (51.1) | 153 (2.4) |
| Information and Communication Systems | 314 (57.0) | 223 (40.5) | 9 (1.6) |
| Agriculture, forestry, and fisheries | 16 (51.6) | 10 (32.3) | 0 (0) |
| Transportation | 33 (48.5) | 36 (52.9) | 1 (1.5) |
| Student | 351 (61.5) | 453 (79.3) | 11 (1.9) |
| Manufacturer | 204 (53.7) | 166 (43.7) | 4 (1.1) |
| Government | 160 (53.5) | 135 (43.7) | 6 (2.0) |
| Unemployed | 431 (53.9) | 343 (42.9) | 15 (1.9) |
| Part-time | 236 (57.3) | 248 (60.2) | 3 (0.7) * |
| Finance and insurance | 66 (54.5) | 52 (43.0) | 5 (4.1) |
| Sales and wholesale | 163 (56.4) | 145 (50.2) | 6 (2.1) |
| Infrastructure and construction | 83 (55.7) | 60 (40.3) | 2 (1.3) |
| Food and beverage and accommodation | 50 (64.1) | 48 (61.5) | 1 (1.3) |
| Other | 254 (54.0) | 228 (48.5) | 10 (2.1) |
| Entertainment and arts | 212 (59.6) | 216 (60.7) | 7 (2.0) |
| Nursing care and welfare | 212 (62.0) | 164 (48.0) | 8 (2.3) |
| Education and research | 358 (54.3) | 401 (60.8) | 14 (2.1) |
| Medical and health | 587 (69.1) | 352 (41.5) | 51 (6.0) |
